# Acetate Improves the Killing of *Streptococcus pneumoniae* by Alveolar Macrophages *via* NLRP3 Inflammasome and Glycolysis-HIF-1α Axis

**DOI:** 10.3389/fimmu.2022.773261

**Published:** 2022-01-20

**Authors:** Marina Gomes Machado, Thiago Andrade Patente, Yves Rouillé, Severine Heumel, Eliza Mathias Melo, Lucie Deruyter, Benoit Pourcet, Valentin Sencio, Mauro Martins Teixeira, François Trottein

**Affiliations:** ^1^ Univ. Lille, CNRS, Inserm, CHU Lille, Institut Pasteur de Lille, U1019 - UMR 9017 - CIIL - Center for Infection and Immunity of Lille, Lille, France; ^2^ Centre National de la Recherche Scientifique, UMR 9017, Lille, France; ^3^ Institut National de la Santé et de la Recherche Médicale U1019, Lille, France; ^4^ Centre Hospitalier Universitaire de Lille, Lille, France; ^5^ Institut Pasteur de Lille, Lille, France; ^6^ Department of Biochemistry and Immunology, Federal University of Minas Gerais, Belo Horizonte, Brazil; ^7^ Department of Parasitology, Leiden University Medical Center, Leiden, Netherlands; ^8^ Institut National de la Santé et de la Recherche Médicale U1011, Lille, France; ^9^ Univ. Lille, U1011 – European Genomic Institute for Diabetes EGID, Lille, France

**Keywords:** alveolar macrophages, short chain fatty acid, immunometabolism, *Streptococcus pneumoniae*, innate immunity, nitric oxide, IL-1β

## Abstract

Short-chain fatty acids (SCFAs) are metabolites produced mainly by the gut microbiota with a known role in immune regulation. Acetate, the major SCFA, is described to disseminate to distal organs such as lungs where it can arm sentinel cells, including alveolar macrophages, to fight against bacterial intruders. In the current study, we explored mechanisms through which acetate boosts macrophages to enhance their bactericidal activity. RNA sequencing analyses show that acetate triggers a transcriptomic program in macrophages evoking changes in metabolic process and immune effector outputs, including nitric oxide (NO) production. In addition, acetate enhances the killing activity of macrophages towards *Streptococcus pneumoniae* in an NO-dependent manner. Mechanistically, acetate improves IL-1β production by bacteria-conditioned macrophages and the latter acts in an autocrine manner to promote NO production. Strikingly, acetate-triggered IL-1β production was neither dependent of its cell surface receptor free-fatty acid receptor 2, nor of the enzymes responsible for its metabolism, namely acetyl-CoA synthetases 1 and 2. We found that IL-1β production by acetate relies on NLRP3 inflammasome and activation of HIF-1α, the latter being triggered by enhanced glycolysis. In conclusion, we unravel a new mechanism through which acetate reinforces the bactericidal activity of alveolar macrophages.

## Introduction

Host metabolites and metabolites emanating from the gut microbiota are well known to play a key role in immunity (1). Among them, short chain fatty acids (SCFAs) are viewed as important regulators of host defense and inflammation in the context of infections (2). The SCFA acetate, propionate and butyrate mostly originate from the fermentation of dietary fibers by the gut microbiota (3, 4). Although the gut microbiota is the main source of SCFAs, acetate can also be produced by eukaryotic cells under specific circumstances such as stress (5). SCFAs have many physiological functions in the gut, like the regulation of mucosal immune response, the maintenance of epithelial barrier and the stimulation of mucous production (4, 6). They also serve as a source of energy for colonocytes (7). Besides being in higher concentrations in the gut, SCFAs can spread through the blood stream and reach distal organs such as the lungs, where they exert immunomodulatory functions (8, 9).

Acetate, the major SCFA produced by the gut microbiota, can signal from the extracellular compartment to the cytoplasm by activating the G protein-coupled receptor free-fatty acid receptor 2 (FFAR2) (10). Alternatively, acetate can also act from the intracellular compartment. It can enter in the cell *via* monocarboxylate transporters (MTCs) or aquaporins and then be converted in acetyl-CoA by acetyl-coenzyme A synthetase (ACSS1) in the mitochondria or by ACSS2 in the cytoplasm. After conversion, acetyl-CoA can enter in the tricarboxylic acid (TCA) cycle serving as metabolic fuel for oxidative phosphorylation (11). In addition, acetyl-CoA in the nucleus can lead to acetylation of histones and consequently modulate gene expression (12). Recently, it was described that acetate can directly bind to glutaminase and enhance its activity, suggesting a new role for acetate in regulating metabolism and immune response (13).

A growing body of evidence points acetate as potential treatment to improve host defense, especially in the context of pulmonary viral and bacterial infections (14–16). Acetate protects mice from respiratory syncytial virus infection by activating alveolar macrophages, which then produce more IFN-β leading to reduced viral loads and inflammation (14, 17). Galvão and colleagues demonstrated that acetate boosts pulmonary defense against *Klebsiella pneumoniae* by increasing the phagocytic ability of neutrophils and the bacterial clearance ability of alveolar macrophages (15). Our group observed that acetate supplementation protects mice against secondary pneumococcal infection in the context of influenza infection, which is characterized by a marked drop in gut microbiota SCFA production (16). This protection is mediated by alveolar macrophages; however, the mode of action of acetate on macrophage’s defense remains elusive.

In the current study, we aimed to better understand the mode of action of acetate on the anti-bacterial activity of macrophage. Our data show that acetate enhanced the bactericidal activity of macrophages against *Streptococcus pneumoniae*, known as the main cause of bacterial pneumonia worldwide, due to higher production of nitric oxide (NO). We demonstrated that NO exacerbation was a result of higher levels of IL-1β triggered by acetate. Surprisingly, acetate-induced IL-1β production was neither dependent of FFAR2, nor of the enzymes responsible for its metabolism (ACSS 1/2). We present a novel mechanism for acetate-induced IL-1β production in which acetate increased the glycolytic profile of macrophages resulting in greater HIF-1α activity and NLRP3 inflammasome activation leading to increased IL-1β production and secretion. Collectively, our results revealed a new mode of action of acetate to improve the host defense.

## Materials and Methods

### Mice and Ethics Statement

Specific pathogen-free C57BL/6J mice (7-week-old, male) were purchased from Janvier (Le Genest-St-Isle, France). *Ffar2* ^-/-^ mice (>10 backcrosses) were produced as previously described (18). Mice were maintained in a biosafety level 2 facility in the Animal Resource Centre at the Institut Pasteur de Lille for at least two weeks prior to usage to allow appropriate acclimatation. Mice were fed a standard rodent chow (SAFE A04, SAFE, Augy, France) and had access to water *ad libitium*. All experiments complied with current national and institutional regulations and ethical guidelines (Institut Pasteur de Lille/B59-350009). The protocols were approved by the institutional ethical committee ‘Comité d’Ethique en Experimentation Animale’ (CEEA) 75. Nord Pas-de-Calais. All experiments were approved by the “Education, Research and Innovation Ministry”, France under the registration number APAFIS22304-201910011647335v3.

### Reagents

Sodium acetate, lipopolysaccharides (LPS) from Escherichia coli O111:B4, nigericin sodium salt, potassium chloride (KCl), 4-[[4-Oxo-2-thioxo-3-[[3-(trifluoromethyl)phenyl]methyl]-5-thiazolidinylidene]methyl]benzoic acid (CY-09), 2-deoxy-D-glucose (2DG), Syrosingopine, Etomoxir and NG-Methyl-L-arginine acetate salt (L-NMMA) were purchased from Sigma (Saint Louis, MO). IL-1β and IL-18 recombinant proteins were purchased from Invitrogen (Waltham, MA). Anti-IL-1β neutralizing monoclonal antibody was purchased from Thermo Fisher Scientifics (Waltham, MA). Rabbit monoclonal antibody anti-ACSS2 (D19C6) was purchased from Cell Signaling (Danvers, MA), mouse monoclonal antibody anti-caspase-1 (Casper-1) was purchased from Adipogen (San Diego, CA). Rabbit polyclonal antibody anti-IL-1β was a kind gift from Proteintech (Rosemont, IL). Mouse IgG kappa binding protein conjugated to horseradish peroxidase (HRP) and mouse anti-rabbit IgG-HRP were purchased from Santa Cruz Biotechnology (Dallas, TX).

### Treatments

Sodium acetate 30 mM, KCl 90 mM, IL-1β recombinant protein 800 pg/mL, anti-IL-1β monoclonal antibody 1.25 µM, IL-18 recombinant protein 2000 pg/mL CY-09 30 µM, L-NMMA 500 µM, Syrosingopine, Etomoxir, and 2-DG were diluted/dissolved in complete RPMI medium. All treatments were added to the cells 1h before stimulation with *S. pneumoniae*, except for L-NMMA which was added at the same time as *S. pneumoniae* infection.

### Infection, Treatment, and Assessment of Bacterial Loads

To assess the role of acetate in host defense, mice were treated with acetate (200 mM, drinking water) five days before the *S. pneumoniae* challenge. Then, mice were anesthetized by intramuscular injection of 1.25 mg of ketamine and 0.25 mg of xylazine, and then intranasally (i.n.) infected with 50 µl of PBS containing 1x10^6^ colony forming units (c.f.u.) of *S. pneumoniae* serotype 1, a serotype linked to invasive pneumococcal disease (clinical isolate E1586). Bacteria in the lungs were counted 30 h after the *S. pneumoniae* challenge by plating serial 10-fold dilutions of lung homogenates onto blood agar plates. The plates were incubated at 37°C with 5% CO_2_ overnight and viable bacteria were counted.

### Alveolar Macrophages Collection and Expansion

C57BL/6J and *Ffar2* ^-/-^ mice were euthanized with 460 mg/kg of pentobarbital sodium, the trachea was exposed, to insert an 18 G cannula. The bronchoalveolar lavage was performed with 1 mL of warm PBS 0.5% FBS 2 mM EDTA, this procedure was repeated 10 times and the lavage was added to a tube containing RPMI 10% FBS. Later, cells were centrifuged, counted, and 1.1x10^6^ cells were plated in 96 mm petri dishes containing 10 mL of RPMI Glutamax (Gibco - Waltham, MA) with 10% FBS (Dominique Dutscher - Bernolsheim, France), 1% Penicillin/streptomycin (Gibco - Waltham, MA) and 5 ng/mL GM-CSF (Peprotech -Cranbury, NJ). After 24 h of incubation, fresh media was added. On day 3, media was removed and centrifuged to keep non-adherent cells, and a fresh media was added until day 6 (19). Cells were removed from the plate, centrifuged, counted, and added to 96 well plates at 1.5x10^6^ cells/mL, where they were stimulated with *S. pneumoniae* in the presence or not of acetate.

### Cell Culture and *In Vitro* Experiments

Max Plank Institute (MPI) cells are self-renewing and non-transformed cells originated from fetal liver of C57BL/6J mouse. MPI cells were used as a model of alveolar macrophages, due to their closer profile (20). Cells were cultivated in RPMI Glutamax with 10% FBS, 1% Penicillin/streptomycin, and 30 ng/mL GM-CSF and incubated on 37°C 5% CO_2_. MPI cells were used from passage 6 until passage 30 and they were negative for mycoplasma contamination, which was assessed with MycoAlert™ Mycoplasma Detection Kit (Lonza - Basel, Switzerland). For all experiments 1.5x10^6^ cells/mL were plated in the absence of GM-CSF and incubated for 3 h or overnight for adhesion. Acetate 30 mM was added 1h prior to stimulation with bacteria. Except for the killing assay, in which live bacteria were used at MOI of 10, all *in vitro* experiments were done with heat-killed *S. pneumoniae* at MOI of 30.

### Nitrite Quantification

Supernatant from MPI, *Acss1* ^-/-^, *Acss2* ^-/-^, and *Acss1/2* ^-/-^ cells previously incubated with heat-killed *S. pneumoniae* MOI 30 in the presence or not of acetate, IL-1β, IL-18, 2-DG 10 mM and CY-09 was collected at indicated time points for nitrite quantification using the Griess Reagent Kit following the protocol from Life Technologies (Carlsbad, CA).

### Killing Assay

MPI, *Acss1* ^-/-^, *Acss2* ^-/-^, and *Acss1/2* ^-/-^ cells and WT and *Ffar2* ^-/-^ alveolar macrophages were previously incubated with heat-killed *S. pneumoniae* MOI 30 in the presence or not of acetate, 2-DG and CY-09 for 24 h, a time point in which only acetate treated cells produce significant concentrations of nitrite to induce bacterial killing (**Figure 2D**). Later, killing assay was performed as described (16, 21). Briefly live and opsonized *S. pneumoniae* MOI 10 was added to the cells in the presence or not of acetate and L-NMMA (NO inhibitor), 2-DG and CY-09 incubated for 1 h at 4°C, for bacterial attachment and then at 2 h 30 min at 37°C for internalization. At this point cells were incubated with Penicillin/Streptomycin 40 U/mL for 30 min to eliminate extracellular bacteria. Then, antibiotics were removed, and cells were incubated for 2 h to allow intracellular bacterial killing. To quantify intracellular viable bacteria left, cells were lysed, diluted, and plated in blood agar plate. We considered the vehicle as having 100% of viable bacteria left, and then, we calculated the % of viable bacterial left for other groups over the vehicle.

### ELISA

Cytokine production was measured from the supernatant of cells after 24 h of stimulation, accordingly to protocol’s manufactures for IL-1β, IL-6, IL-12p40, IL-18 (Invitrogen - Waltham, MA) and TNF-α (R&D Systems - Minneapolis, MN).

### Acetate Quantification

Acetate concentration was measured from the supernatant of cells after 24 h of stimulation, using the Acetate Colorimetric Assay Kit accordingly to the protocol’s manufactures Sigma (Saint Louis, MO).

### RNA Extraction, RNAseq, qRT-PCR

Total RNA from cellular lysate was extracted using the NucleoSpin^®^ RNA kit (Macherey−Nagel - Hoerdt, Germany). RNA was used to generate cDNA with High-Capacity cDNA Archive Kit (Life Technologies - Carlsbad, CA). Later, the cDNA was mixed with SYBR Green (Thermo Fisher Scientific - Waltham, MA) based real-time PCR and amplified for detection on QuantStudio 12K Flex Real-Time PCR Systems (Applied Biosystems - Waltham, MA) according to manufacturer’s protocol. Specific primers were generated using Primer Blast and ordered at Eurofins Scientifics (Luxembourg) (**Table 1**). The expression of all genes was normalized with the housekeeping gene TATA-Box Binding Protein (*Tbp)* and the fold increase was calculated over the control group. To assess gene expression profile by RNA-sequencing, extracted RNA was send to Nice-Sophia-Antipolis Functional Genomics Platform, France. Next generation sequencing was performed on Illumina NextSeq500 (Illumina - San Diego, CA). The obtained libraries of sequences (reads) were aligned with STAR on the mm10 genome version during the primary analysis. Secondary analysis was done with STAR aligner RNA-seq pipeline. In total, 14501 genes were included in the analysis, with at least 20 reads each. The data was normalized over total gene expression. RNA sequencing raw data and processed data were deposited in GEO at NCBI https://www.ncbi.nlm.nih.gov/geo/query/acc.cgi?acc=GSE183089 Accession number: GSE183089.

**Table 1:** Primer sequences used for PCR.

*Tbp* F - GGCGGTTTGGCTAGGTTTCT *Tbp* R - TGCCGTAAGGCATCATTGGA	*Pfkfb3* F - CCAGAGCCGGGTACAGAAGA *Pfkfb3* R - GAGGCCACAACAGTAGGGTC
*Slc2a4* F - CAGATCGGCTCTGACGATGG *Slc2a4* R - GCCACGTTGCATTGTAGCTC	*Pgk1* F - CGAGCCTCACTGTCCAAACT *Pgk1* R - TCTGTGGCAGATTCACACCC
*Serpine1* F - GTCGTGGAACTGCCCTACC *Serpine1* R - GCGTCTCTTCCCACTGTCAA	*Aldoa* F - CGCTCCTTAGTCCTTTCGCC *Aldoa* R - AATGCAGGGATTCACACGGT
*Pdk1* F - CCACTGAGGAAGATCGACAGAC *Pdk1* R - AGAGGCGTGATATGGGCAATCC	*Vegfa* F - GCAGCTTGAGTTAAACGAACG *Vegfa* R - GGTTCCCGAAACCCTGAG
*Ucp3* F - ACCCGATACATGAACGCTCC *Ucp3* R - TCATCACGTTCCAAGCTCCC	*Inos* F - CAGCTGGGCTGTACAAACCTT *Inos* R - CATTGGAAGTGAAGCGTTTCG
*Il-1b* F - TCGTGCTGTCGGACCCATA *Il-1b* R - GTCGTTGCTTGGTTCTCCTTGT	*Casp1* F - ACAAGGCACGGGACCTATG *Casp1* R - TCCCAGTCAGTCCTGGAAATG
*Nlrp3* F - ATTACCCGCCCGAGAAAGG *Nlrp3* R - TCGCAGCAAAGATCCACACAG	*Asc* F - CTTGTCAGGGGATGAACTCAAAA *Asc* R - GCCATACGACTCCAGATAGTAGC
*Mct1* F - TGTGTGGAAAACCTACCGGG *Mct1* R - TGCCAACCACTCCCTACCTA	*Mct4* F - GGCGGTAACAGGTGAAAGCA *Mct4* R - ATAGGGCGACGCTTGTTGAA
*Acss1* F - GTTTGGGACACTCCTTACCATAC *Acss1* R - AGGCAGTTGACAGACACATTC	*Acss2* F - TGCCACCATAAGTCAACCCC *Acss2* R - ACAGGGCATTCAGAAGGGTG
*Ffar2* F - TTAATCTGACCCTGGCGGAC *Ffar2* R - AGC GCGCACACGATCTTT	*Hif1a* F - ACCTTCATCGGAAACTCCAAA *Hif1a* R - ACTGTTAGGCTCAGGTGAACT

### Knockout With CRISPR Cas9

Knockout cells were generated following the protocol published by Ann Ran and colleagues (22). Briefly, ACSS1 and ACSS2 guide RNAs were designed using the Gecko2 Mouse library. Six target sequences were designed for each gene, to further allow the selection of the best knockout (**Table 2**). Annealed oligonucleotide pairs containing a guide RNA-coding sequence were inserted in the BsmBI sites of Lenti CRISPR v2 (Addgene #52961) or Lenti CRISPR v2-blast (Addgene #83480) plasmids containing a resistance gene for puromycin or blasticidin, respectively. Lentiviral particles were produced by transient cotransfection of 293TT cells with a guide RNA-carrying lentiCRISPRv2 or lentiCRISPRv2-blast plasmid, a packaging vector (psPAX2, Addgene #12260), and a vector expressing the vesicular stomatitis virus glycoprotein (VSV-G), using Turbofect as a transfection reagent according to the manufacturer’s protocol (Thermo Fisher Scientifics). Control lentiviruses were generated using lentiCRISPRv2 or lentiCRISPRv2-blast plasmid with no guide RNA coding sequence inserted. Transfected cells were incubated for 3 days at 33°C. Cell culture supernatants containing the lentiviral particles were collected, passed through 0.45 µm filters and stored at -80°C. Later, lentiviruses were added to MPI cells at 30% of confluence for 24 h. After two weeks of antibiotics selection (puromycin 7 µg/mL or blasticidin 1 µg/mL), cells were expanded to have the KO gene assessed by RT-PCR and Western Blot. As ACSS1 is low expressed, we could not detect it by western blot. KO cells with a reduction higher than 90% of mRNA expression were kept in culture for further experiments.

**Table 2:** Guide RNA sequences.

*Acss1*.1 5’-CACCGAAGAGACATGGAGTGCACCG-3’ 5’-AAACCGGTGCACTCCATGTCTCTTC-3’	*Acss2*.1 5’-CACCGAAACATCTGCTACAACGTGC-3’ 5’-AAACGCACGTTGTAGCAGATGTTTC-3’
*Acss1*.2 5’-CACCGGCTCCTACCTTGTGCGTCA-3’ 5’-AAACTGACGCACAAGGTAGGAGCC-3’	*Acss2*.2 5’-CACCGTACTGGAAAACCGCATGCCC-3’ 5’-AAACGGGCATGCGGTTTTCCAGTAC-3’
*Acss1*.3 5’-CACCGGCTCACAGGACGGACACCA-3’ 5’-AAACTGGTGTCCGTCCTGTGAGCC-3’	*Acss2*.3 5’-CACCGACCACAAGTTCCAAGATCAT-3’ 5’-AAACATGATCTTGGAACTTGTGGTC-3’
*Acss1*.4 5’-CACCGTCTGGAGACCACATGCCGCC-3’ 5’-AAACGGCGGCATGTGGTCTCCAGAC-3’	*Acss2*.4 5’-CACCGGTCACCTGTAGTGATGAGC-3’ 5’-AAACGCTCATCACTACAGGTGACC-3’
*Acss1*.5 5’-CACCGTATGCCGCCATGACGCACA-3’ 5’-AAACTGTGCGTCATGGCGGCATAC-3’	*Acss2*.5 5’-CACCGATCACATACCGTGAACTCC-3’ 5’-AAACGGAGTTCACGGTATGTGATC-3’
*Acss1*.6 5’-CACCGTCTGGATATCCCCCTTGAAC-3’ 5’-AAACGTTCAAGGGGGATATCCAGAC-3’	*Acss2*.6 5’-CACCGCAGCAATGTTCTCCGTAAAC-3’ 5’-AAACGTTTACGGAGAACATTGCTGC-3’

### Knockdown With siRNA

ON-TARGETplus siRNA (SMARTpool) for HIF-1α 20 µM (Dharmacon - Lafayette, CO) or scramble siRNA 20 µM (Eurofins Scientifics) were mixed with PBS and lipofectamine RNAiMAX (Thermo Fisher Scientifics) for 30 minutes, then MPI cells (1.5x10^5^ cells/mL) in RPMI were added to the mixture and incubated at 37 °C and 5% CO_2_ for 24 h. Later, cells were washed with fresh medium and incubated for 3 h. Medium was then removed, and cells were pre-treated or not with acetate 30 mM for 1 h, followed by *S. pneumoniae* stimulation during 18 h. Supernatant was collected for ELISA and cells were collected for qRT-PCR.

### Western Blot

Cellular extraction was done with RIPA buffer (100 µl/10^6^ cells). Then, cellular lysate was centrifuged at 10000 g for 10 min, the supernatant was collected and equal amounts of protein were added to Laemmli buffer (EcoTech Biotechnology - Istanbul, Turkey) to a final concentration of 1x and boiled at 95°C for 5 min. Western blot from supernatant was done as described (23). Briefly, supernatant was collected and precipitated with equal volume of methanol and 0.25 volumes of chloroform. Then it was centrifuged at 20000 g for 10 min, the first phase was discarded, 500 µl of methanol was added and centrifuged again. Later, the supernatant was removed, the pellet dried, resuspended in Laemmli buffer 1x and boiled at 95°C for 5 min. Protein samples were loaded in SDS-PAGE stain free gel (Bio-Rad Laboratories - Hercules, CA). Then, after UV light exposure, total loaded protein was assessed by total lane density on ChemiDoc MP System (Bio-Rad). Primary antibodies were used at 1:1000 and secondary antibodies at 1:3000. Image of PVC membrane was also acquired with ChemiDoc MP systems, which was used for bands quantification. In all western blot data, the band was normalized by total lane density from each sample and results are represented as fold increase over the control.

### Metabolic Analysis

MPI cells (1x10^6^ cells/mL) were seeded in complete RPMI for 3 h and then pre-treated with acetate 30 mM for 1 h followed by *S. pneumoniae* stimulation. After 24 h, cells were washed with Seahorse RPMI medium (Agilent - Santa Clara, CA) supplemented with glutamine 2 mM and/or glucose 10 mM and incubated for 1 h at 37°C without CO_2_. Extracellular acidification rate (ECAR) and oxygen consumption rate (OCR) were measured on Agilent Seahorse XFe96 Analyzers upon injection of different drugs combination. For glycolytic stress analysis we used: Port A: Glucose 10 mM (Agilent), Port B: Oligomycin (Oligo) 1 µM (Cayman - Ann Arbor, MI), Port C: 2-Deoxy-D-Glucose (2-DG) 50 mM (Sigma). For mitochondrial stress analysis we used: Port A: Oligomycin (Oligo) 1 µM (Cayman), Port B: Carbonyl cyanide 4-(trifluoromethoxy)phenylhydrazone (FCCP) 1.5 µM (Sigma), Port C: Rotenone and Antimycin (R/A) 1 µM (Sigma). After ECAR and OCR measurements, total protein was assessed from cellular lysates and used to normalized ECAR and OCR data. To calculate glycolysis, we subtracted the highest measure after glucose injection by the last basal measure, for each group. To calculate basal respiration, we subtracted the lower measurement after R/A injection from the last measurement of basal respiration. For maximal respiration we subtracted the lower measurement after R/A injection from the maximal measurement after FCCP injection. For spare respiratory capacity we subtracted the lower measurement after R/A injection, and the last measurement of basal respiration from the maximal measurement after FCCP injection.

### Statistical Analysis

Results are expressed as the mean with standard error of the mean (SEM) unless otherwise stated. The statistical analyses were done using R software (RNA-Seq) or GraphPad Prism v8 software. For each analysis, at least three independent experiments were performed. Due to the variation led by the infection, we show here a representative data from the three independent experiments. All experiments were done in triplicates unless otherwise stated. Normality test was done for all results to determine the usage of parametric or non-parametric tests. Data containing two groups with normal distribution were analyzed with Unpaired Student’s t-test, and the nonparametric ones were analyzed with Mann-Whitney U test. When more than two factors were present in the analysis Two-way ANOVA test followed by Sidak’s multiple comparisons test was performed. Statistical details of experiments can be found in the figure legends. For RNA-Seq, normalization and differential analysis were performed using the DESeq2 package in R. Log2 fold change was calculated over *S. pneumoniae* stimulated cells without acetate treatment. Volcano plot represents genes with log2 fold change > 0.6 and p-adjunctive < 0.05 were considered as up-regulated and genes with log2 fold change < -0.6 and p-adjunctive < 0.05 were considered as down-regulated. Enrichment analysis was done in the Metascape platform, for Gene Ontology (GO) biological process. In the first analysis it was included all genes with p-adjunctive < 0.05, and fold change > 1.5. In the second analysis it was included all genes with p-adjunctive < 0.05, and fold change < -1.5. The pathway enrichment had a p-value cutoff of 0.01 and minimum enrichment of 1.5. The raw data was analyzed and pathways that were redundant or related to other cellular type were excluded. Then, three graphics were done, one containing all enriched process by acetate, other with 10 pathways up-regulated by acetate associated to immunological and metabolic processes and the last with 10 pathways downregulated by acetate, also associated to immunological and metabolic processes.

## Results

### Acetate Enhances the Bactericidal Activity of Alveolar Macrophages Through NO Production

**Figure 1:**
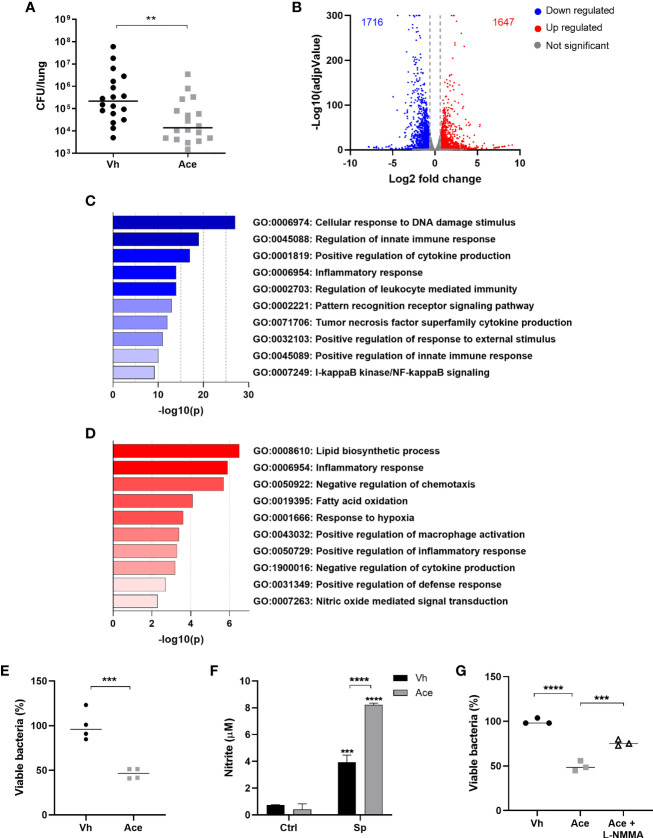
Acetate treatment improves the killing ability of macrophages by increasing NO production. **(A)** Bacterial quantification in the lungs from C57BL/6J mice treated with acetate (200 mM in drinking water) for 5 days, intranasally infected with *S. pneumoniae* (1x10^6^ CFU) and euthanized 30 h post-infection. Pool of three independent experiments (n=6 per experiment). **(B)** Volcano plot from MPI cells pre-treated or not with acetate, stimulated with *S. pneumoniae* and lysed after 18 h for RNA extraction and sequencing. Fold change was done over vehicle-treated cells stimulated with *S. pneumoniae*. Normalization and differential analysis were performed using the DESeq2 package in R. Genes with log2 fold change > 0.6 and p-adjunctive < 0.05 were considered as up-regulated and genes with log2 fold change < -0.6 and p-adjunctive < 0.05 were considered as down-regulated. **(C)** Enrichment analysis from MPI cells pre-treated or not with acetate, stimulated with *S. pneumoniae* and lysed after 18 h for RNA extraction and sequencing. Enrichment analyses were done over acetate stimulated cells in the Metascape platform. The figure shows selected pathways downregulated by acetate. **(D)** Enrichment analysis from MPI cells pre-treated or not with acetate, stimulated with *S. pneumoniae* and lysed after 18 h for RNA extraction and sequencing. Enrichment analyses were done over vehicle stimulated cells in the Metascape platform. The figure shows selected pathways enriched by acetate. **(E, G)** % of intracellular viable bacteria left 6 h post infection of activated MPI cells treated or not with acetate and/or L-NMMA, normalized to vehicle group. **(F)** Nitrite concentration assessed by Griess assay from supernatant of MPI cells pre-treated or not with acetate and stimulated or not with *S. pneumoniae* during 48 h. Data showing the median **(A, E, G)** or bars showing the mean and error showing the SEM **(F)** of quadruplicates. Results are representative of three independent experiments. Statistical analysis was done using Mann-Whitney U test **(A)**, Unpaired Student’s t-test **(E)**, Two-way ANOVA corrected with Sidak’s multiple comparisons test **(F)** and One-Way ANOVA **(G)** (**p < 0.01, ***p < 0.001, ****p < 0.0001).

Prior findings suggest that acetate can exert protective effect against respiratory bacteria including *K. pneumoniae* and *S. pneumoniae*, albeit for the later this was studied in a context of prior influenza infection (15, 16). To assess the potential effectiveness of acetate against pneumococcal infection alone, we evaluated the bacterial burden in mice receiving acetate supplementation (drinking water). It was observed that acetate supplementation diminished bacterial counts in the lungs (**Figure 1A**). Interestingly, several studies demonstrated that acetate can target alveolar macrophages - the main sentinel cells of the pulmonary immune system - to modulate their activity (15–17). Nevertheless, the underlying mechanisms are still elusive. To uncover this mechanism of action, we used a well-described alveolar macrophage cell line (termed MPI), that mimics the main feature of primary alveolar macrophages (20). Importantly, RT-PCR analysis indicated that MPI cells and primary alveolar macrophages express genes involved in acetate recognition (*Ffar2*), transport (*Mct1* and *Mct4*) and metabolism (*Acss2* and *Acss1*), albeit at different levels (**Figure S1**).

We first determined the impact of acetate supplementation on gene expression profile. To this end, macrophages were exposed to acetate or vehicle and then stimulated with *S. pneumoniae*. Gene expression analysis by RNA-sequencing indicated that despite the known ability of acetate to enhance histone acetylation - a process that favors gene expression - the numbers of up-regulated and down-regulated genes were similar between vehicle-treated cells and acetate-treated cells (**Figure 1B**). Enrichment analysis of transcriptomic datasets highlighted the downregulation of several genes related to inflammation (cytokine production) and NF-κB signaling pathways (**Figure 1C**). On the other hand, processes - with a high number of upregulated genes - related to cellular organization, locomotion, metabolic process, response to stimulus and immune response were evidenced in response to acetate (**Figure S2**). Among enriched metabolic process, acetate enhanced lipid biosynthetic process and fatty acid oxidation, mechanisms known to be involved in the metabolism of acetate (**Figure 1D**). Acetate also increased response to hypoxia and induced a signature evoking macrophage activation and defense response. Interestingly, NO-mediated signal transduction was also enhanced, a pathway known to play a role in intracellular bacterial killing (24, 25). Altogether, this gene expression profile suggested a role for acetate in bactericidal activity of macrophages. In order to confirm this hypothesis, we assessed the effect of acetate treatment on the intracellular killing ability of macrophages *in vitro*. Consistently, macrophages activated in the presence of acetate displayed less viable bacteria, reflecting a better ability to kill *S. pneumoniae* relative to vehicle-treated cells (**Figure 1E**). As NO is the major reactive specie involved in *S. pneumoniae* killing (24, 26), we assessed its production upon acetate treatment. Of interest, compared to the vehicle, acetate treatment resulted in a higher production of NO by macrophages, as assessed by nitrite measurement (**Figure 1F**). It should be noted that acetate alone failed to induce NO production, indicating that acetate likely enhanced the NO production triggered by *S. pneumoniae* rather than inducing it by itself. To investigate the potential link between acetate, NO and bacterial killing, the killing assay was performed in the presence of a NO inhibitor. As depicted in **Figure 1G**, the blockade of NO production reduced the effectiveness of acetate on bacterial killing. Collectively, these results suggest that acetate promotes bacterial killing by alveolar macrophages through NO production.

### Acetate Increases NO Production *via* IL-1β

**Figure 2:**
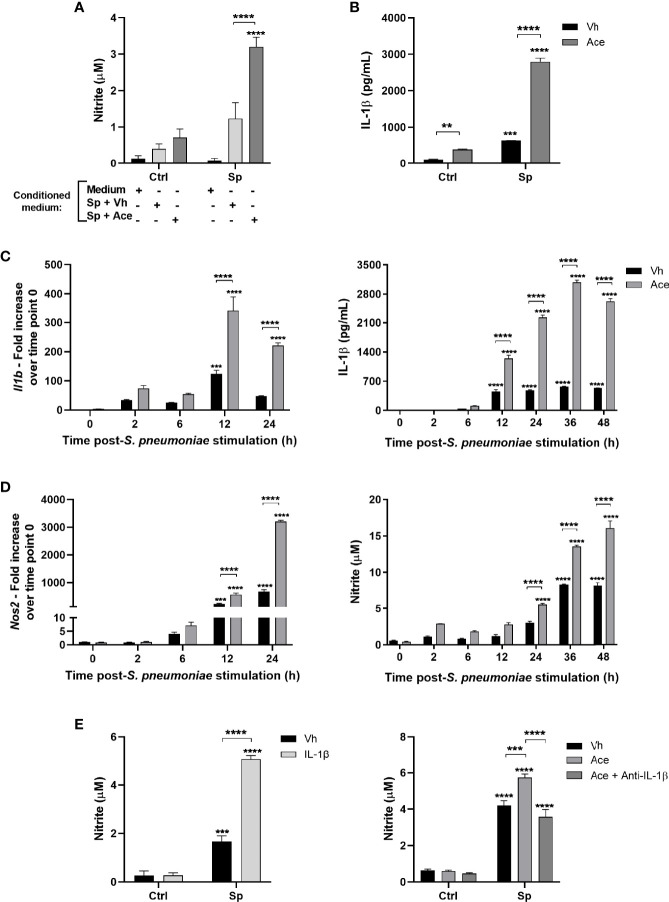
Acetate increases nitric oxide production *via* IL-1β. **(A)** Nitrite concentration assessed by Griess assay from supernatant of MPI cells pre-treated with conditioned medium, then stimulated or not with *S. pneumoniae.* Conditioned medium is the supernatant of MPI cells stimulated with *S. pneumoniae* in the presence or absence of acetate during 24 h. **(B)** ELISA of IL-1β from supernatant of MPI cells pre-treated or not with acetate and stimulated or not with *S. pneumoniae* during 24 h. **(C, D)** Kinetics from MPI cells pre-treated with acetate and then stimulated with *S. pneumoniae*. Cellular lysate was used for *Il1b* and *Nos2* mRNA quantification by RT-PCR, and supernatants for IL-1β secretion by ELISA and nitrite by Griess assay. For RT-PCR fold increase was calculated over time point 0 (Vh). **(E)** Nitrite concentration assessed by Griess assay from supernatant of MPI cells: Pre-treated or not with IL-1β and stimulated or not with *S. pneumoniae* during 24 h **(E)** *left* panel). Pre-treated or not with acetate and/or anti-IL-1β antibody and stimulated of not with *S. pneumoniae* for 48 h **(E)** *right* panel). Bars showing the mean and error showing the SEM of triplicates. Results are representative of three independent experiments. Statistical analysis was done using Two-way ANOVA corrected with Sidak’s multiple comparisons test (**p < 0.01, ***p < 0.001, ****p < 0.0001).

To better understand the mode of action of acetate, we investigated whether the effect of acetate on NO production was direct or indirect. For this, conditioned media from vehicle-treated cells or acetate-treated cells were added to macrophages in the presence or absence of *S. pneumoniae*. Of note both conditioned media had low concentrations of acetate (**Figure S3**). The conditioned medium originated from acetate-treated cells induced higher production of nitrite when compared to conditioned medium from vehicle-treated cells, suggesting that factor(s) produced by treated cells lead to NO production (**Figure 2A**). It is known that pro-inflammatory cytokines such as IFN-γ, TNF-α and IL-1β can improve NO production by different cell types (27–29). Acetate treatment resulted in a marked increase of IL-1β production by alveolar macrophages (**Figure 2B**). In line with the pathways downregulated by acetate (*e.g.* NF-κB signaling) observed on enrichment analysis (**Figure 1C**), acetate reduced the production of TNF-α and IL-12p40, while no significant effect was seen on IL-6 production (**Figure S4A**). These data were confirmed using primary alveolar macrophages (**Figure S4B**).

To evaluate the potential link between IL-1β and NO production in the context of acetate supplementation, we assessed the time course of *Il1b* and *Nos2* gene expression together with IL-1β and nitrite production. Acetate markedly increased *Il1b* gene expression and IL-1β protein levels after 12h of stimulation (**Figure 2C**), meanwhile *Nos2* expression peaked at 24h post-stimulation (**Figure 2D**, *left* panel). In agreement, nitrite levels started to increase after 24h of stimulation (**Figure 2D**, *right* panel). Hence, IL-1β secretion appears to precede NO production indicating a potential role of IL-1β in NO synthesis. To investigate whether IL-1β can boost NO production by macrophages, cells were treated with recombinant IL-1β protein. Indeed, IL-1β treatment enhanced nitrite production by macrophages upon *S. pneumoniae* stimulation (**Figure 2E**, *left* panel). To confirm that NO enhancement by acetate is dependent on IL-1β, cells were treated with anti-IL-1β antibody. IL-1β neutralization abolished the effect of acetate on nitrite production (**Figure 2E**, *right* panel). As IL-1β, IL-18 synthesis can also depend on NLRP3 inflammasome activation (30), we then investigated whether acetate could increase IL-18 production by macrophages. Indeed, acetate enhanced the production of IL-18 in the presence or absence of *S. pneumoniae* (**Figure S5A**). Although many pro-inflammatory cytokines are shown to induce NO production, the potential effect of IL-18 is currently unknown. Thus, we assessed NO production upon addition of recombinant IL-18 and *S. pneumoniae*. Unlike IL-1β, IL-18 had no impact on NO production by macrophages stimulated with *S. pneumoniae *(**Figure S5B**). We conclude that acetate improves IL-1β production by *S. pneumoniae*-conditioned macrophages and IL-1β acts in an autocrine manner to improve NO production.

### IL-1β Production Driven by Acetate Is Independent of FFAR2, ACSS1, and ACSS2

**Figure 3:**
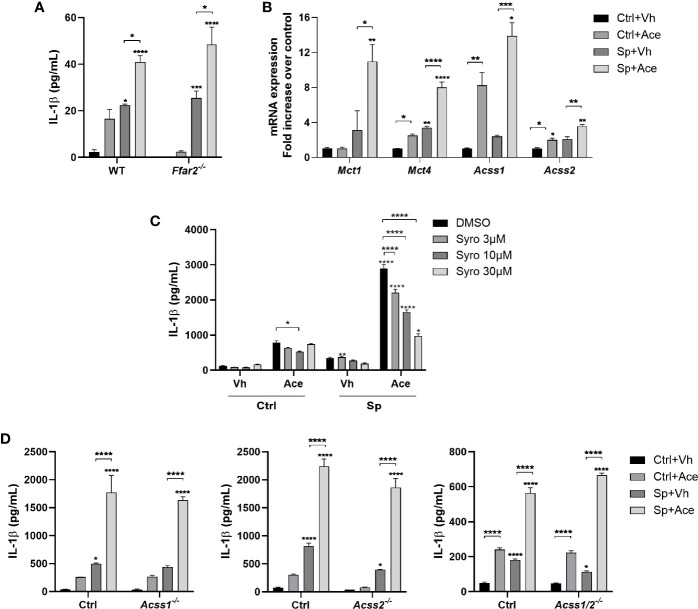
IL-1β production driven by acetate is independent of FFAR2, ACSS1 and ACSS2. **(A)** ELISA for IL-1β from supernatant of alveolar macrophages obtained from WT and *Ffar2* ^-/-^ mice pre-treated or not with acetate, stimulated or not with *S. pneumoniae* for 24 h. **(B)** Fold increase of mRNA from monocarboxylate transporters (*Mct*) and acetyl-CoA synthetase (*Acss*) assessed by RT-PCR from MPI cells pre-treated or not with acetate and stimulated or not with *S. pneumoniae* for 18 h. Fold increase was calculated over control (Ctrl+Vh). **(C, D)** ELISA for IL-1β from supernatant of MPI cells **(C)** or MPI control, *Acss1* ^-/-^, *Acss2* ^-/-^ and *Acss1/2* ^-/-^ cells **(D)** pre-treated or not with acetate and Syrosingopine **(C)** and stimulated or not with *S. pneumoniae* for 24 h. Bars showing the mean and error showing the SEM of triplicates. Results are representative of three independent experiments. Statistical analysis was done using Two-way ANOVA corrected with Tukey’s **(A, D)** or Sidak’s multiple comparisons test **(B, C)** (*p < 0.05, **p < 0.01, ***p < 0.001, ****p < 0.0001).

Due to the important role of IL-1β in NO production and subsequent *S. pneumoniae* killing, we investigated the mechanisms by which IL-1β production was increased upon acetate treatment. As mentioned before, acetate has three main mechanisms of action: (1) it can directly activate FFAR2; it can be converted to Acetyl CoA by ACSS1 or ACSS2, serving as (2) substrate for the cellular metabolism or (3) for acetylation of proteins, especially histones. As, in response to acetate, FFAR2 has been described to increase IL-1β production by neutrophils (15), we addressed whether this was the case for alveolar macrophages. To this end, alveolar macrophages were collected from wild type and *Ffar2^-/-^* mice. As depicted in **Figure 3A**, FFAR2 deficiency did not impact IL-1β production triggered by acetate in the presence of *S. pneumoniae*. In line, FFAR2 deficiency had no impact on the acetate-triggered bacterial killing ability of macrophages (**Figure S6**). Of interest, acetate triggered expression of transcripts encoding the transporters, MCTs and the enzymes responsible for acetate conversion to acetyl CoA, ACSS1 and ACSS2 (**Figure 3B**). To assess the potential role of MCTs in IL-1β production, the MCT1 and MCT4 inhibitor syrosingopine was used. We observed a dose-dependent inhibition of IL-1β production upon acetate treatment (**Figure 3C**). Although this result might indicate that acetate act from the intracellular compartment, MCTs transport other substances such as lactate, and therefore this result could also be a consequence of lactate transport inhibition. To address if this effect was also dependent on the metabolism of acetate, we knocked out ACSS1 and/or ACSS2 enzymes by CRISPR Cas9 technology (**Figures S7A–C**). Removing ACSSs activities, either ACSS1 or ACSS2 or both, did not impact the ability of macrophages to produce IL-1β upon acetate treatment (**Figure 3D**). Additionally, the absence of these enzymes had neither an impact in the NO production nor an impact in the increased killing induced by acetate (**Figures S7D, E**). Thus, the effect of acetate on IL-1β and NO production, and on killing is independent of FFAR2, ACSS1 and ACSS2. Additionally, acetate relies on MCTs to induce IL-1β production.

### Acetate Increases the Secretion of IL-1β *via* NLRP3 Inflammasome

**Figure 4:**
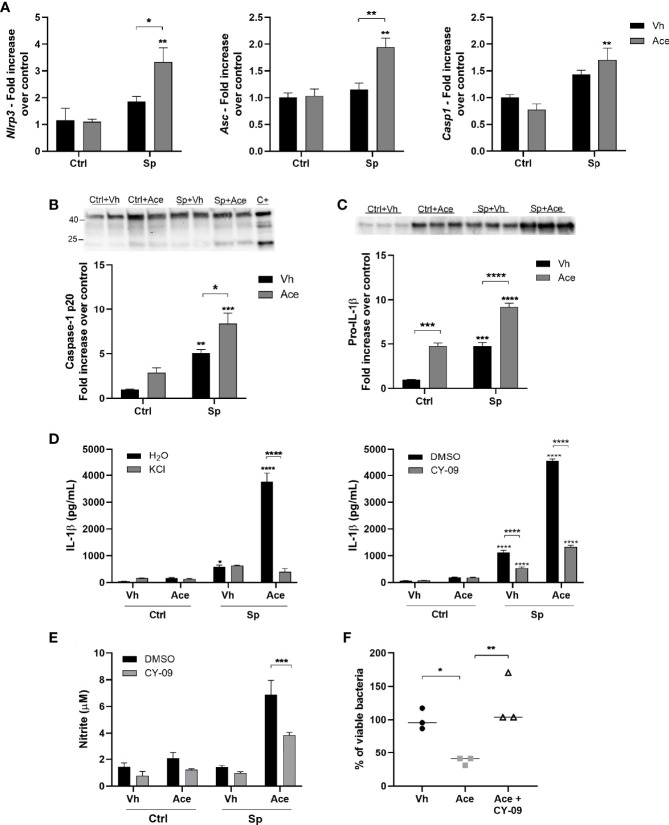
Acetate increases the secretion of IL-1β *via* NLRP3. **(A)** Fold increase of *Nlrp3*, *Asc* and *Casp1* mRNAs assessed by RT-PCR from MPI cells pre-treated or not with acetate and stimulated or not with *S. pneumoniae* for 18 h. Fold increase was calculated over control (Ctrl+Vh). **(B**, **C)** Western blot for Caspase 1 from supernatant and pro-IL-1β from cellular lysate of MPI cells pre-treated or not with acetate, and then incubated or not with *S. pneumoniae* for 24 h. Caspase-1 p20 and pro-IL-1β quantifications were normalized to total lane density (total protein) from stain free gel image. For caspase 1, a positive control was done adding 300 ng/mL of LPS for 4 h, followed by 20 µM of nigericin for 30 min. Western blot for caspase 1 was done in duplicates/triplicate and repeated three times. Graphic shows a pool from the mean of three independent experiments. **(D)** ELISA for IL-1β from supernatants of MPI cells pre-treated or not with acetate, KCl or CY-09 and then stimulated or not with *S. pneumoniae* for 24 h. **(E)** Nitrite concentration assessed by Griess assay from supernatant of MPI cells pre-treated or not with acetate in the presence of absence of CY-09 30 µM and stimulated or not with *S. pneumoniae* during 48 h. **(F)** % of intracellular viable bacteria left 6 h post infection of activated MPI cells treated or not with acetate and/or CY-09 30 µM, normalized to vehicle group. Bars showing the mean and error showing the SEM **(A–E)** and data showing the median **(F)** of triplicates. Results are representative of three independent experiments. Statistical analysis was done using Two-way ANOVA **(A–E)** and One-Way ANOVA **(F)** corrected with Sidak’s multiple comparisons test (*p < 0.05, **p < 0.01, ***p < 0.001, ****p < 0.0001).

Strong evidence shows that acetate is able to modulate NLRP3 inflammasome activity. It was shown that bone-marrow derived macrophages (BMDM) stimulated with LPS and nigericin in the presence of acetate present an attenuated activation of NLRP3 inflammasome (31). On the other hand, neutrophils stimulated with the bacteria *C. difficile* in the presence of acetate presented enhanced NLRP3 activation (32). To investigate the implication of NLRP3 inflammasome in enhanced IL-1β secretion driven by acetate, the expression of NLRP3 inflammasome genes was first assessed. Acetate up-regulated *Nlrp3* and *Asc* gene expression (only in the presence of *S. pneumoniae*) whilst it had no effect on *Casp1* gene expression (**Figure 4A**). We then measured caspase 1 activation by quantifying cleaved caspase 1 p20. As shown in **Figure 4B**, acetate treatment alone or in combination with *S. pneumoniae* enhanced caspase 1 activation. In accordance, pro-IL-1β protein levels were also increased by acetate and even enhanced in the presence of *S. pneumoniae* (**Figure 4C**). To better understand the role of NLRP3 inflammasome in acetate-mediated IL-1β secretion, *S. pneumoniae*-conditioned macrophages were treated with KCl and CY-09, two well described NLRP3 inflammasome inhibitors (33, 34). As depicted in **Figure 4D**, acetate-mediated IL-1β production by *S. pneumoniae*-conditioned macrophages was largely dependent on NLRP3 inflammasome activity. In order to confirm the dependency of NLRP3 activation to NO production and bacterial killing by acetate-treated macrophages, these parameters were evaluated in the presence of CY-09. The blockage of NLRP3 inflammasome reduced nitrite production and bacterial killing induced by acetate (**Figures 4E, F** **)**. Taken together, acetate increases the expression of NLRP3 inflammasome components, NLRP3 and ASC, activates caspase 1 and induces pro-IL-1β production, thus resulting in higher IL-1β secretion, NO production, and bacterial killing ability.

### Acetate Modulates Oxygen Consumption by Macrophages but Fatty Acid Oxidation Is Not Involved in IL-1β Production

As mentioned before, acetate can be converted into acetyl CoA and fuel the TCA cycle and boost mitochondrial respiration (35). To address the impact of acetate on mitochondrial respiration, we assessed the oxygen consumption rate of macrophages pre-treated or not with acetate in the presence or absence of *S. pneumoniae*. We observed that *S. pneumoniae* stimulation, as many pro-inflammatory stimuli, reduced the consumption of oxygen (**Figure S8A**). Acetate treatment had no impact on basal respiration; however, maximal respiration and spare respiratory capacity were increased by acetate in the absence and presence of *S. pneumoniae* (**Figure S8B**). In line with the enrichment analysis (**Figure 1D**), this result indicates that acetate might increase fatty acid oxidation. The later is known to induce IL-1β production by macrophages (36, 37). However, blockage of fatty acid oxidation by etomoxir had no impact on IL-1β production induced by acetate (**Figure S8C**). Hence, the alteration of oxygen consumption by macrophages is not linked to IL-1β production induced by acetate.

### Acetate Enhances Glycolysis in Macrophages Leading to Higher IL-1β Production

**Figure 5:**
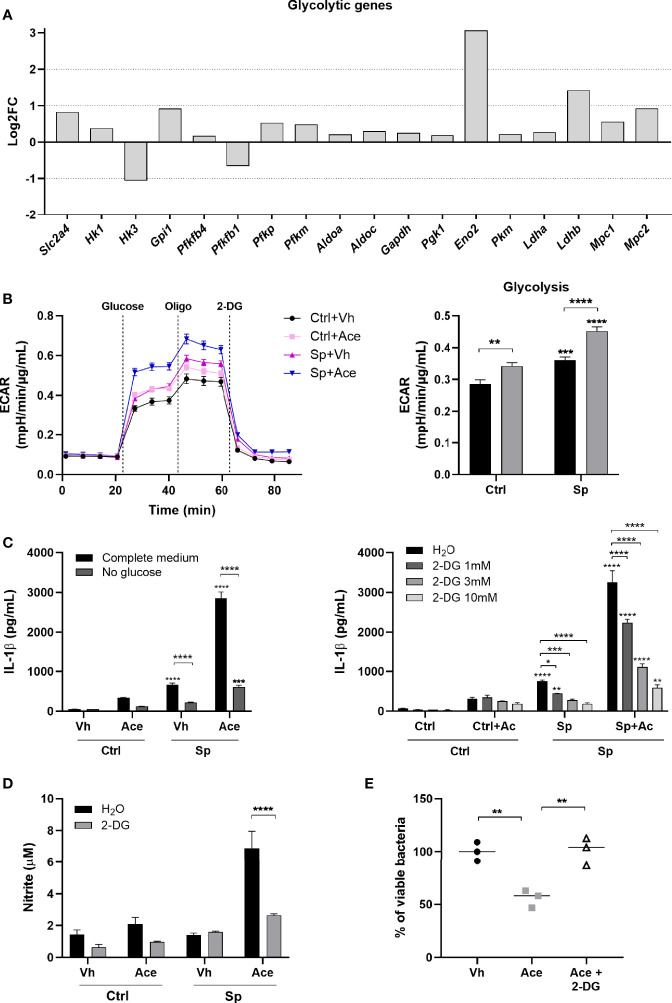
Acetate enhances glycolysis leading to higher IL-1β production. **(A)** Log_2_ fold change of glycolytic genes expression of acetate treated MPI cells stimulated with *S. pneumoniae* over vehicle treated cells stimulated with *S. pneumoniae*. Data were obtained from RNA-Seq. All represented genes had p < 0.01 in the comparison between the two groups. Solute carrier family 2, facilitated glucose transporter member 4 (*Slc2a4*), hexokinase (*Hk1, Hk3*), glucose-6-phosphate isomerase (*Gpi1*), 6-phosphofructo-2-kinase (*Pfkfb4, Pfkfb1*), ATP-dependent 6-phosphofructokinase platelet and muscle types (*Pfkp*, *Pfkm*), fructose-bisphosphate aldolase (*Aldoa, Aldoc*), glyceraldehyde-3-phosphate dehydrogenase (*Gapdh*), phosphoglycerate kinase 1 (*Pgk1*), gamma-enolase (*Eno2*), (*Pkm*), L-lactate dehydrogenase (*Ldha, Ldhb*), mitochondrial pyruvate carrier (*Mpc1, Mpc2*). **(B)** Glycolysis stress assay of MPI cells pre-treated or not with acetate, and then stimulated or not with *S. pneumoniae* for 24 h. ECAR was measured in Seahorse after injection of glucose, oligomycin (Oligo) and 2-Deoxy-D-glucose (2-DG) (*right panel*). Glycolysis was calculated by the subtraction of the highest measure after glucose injection by the last basal measure, for each group (*left panel*). **(C)** ELISA for IL-1β from supernatant of MPI cells pre-treated or not with acetate in the presence or not of glucose (*left panel*) or 2-DG (*right panel*) and then stimulated or not with *S. pneumoniae* for 24 h. **(D)** Nitrite concentration assessed by Griess assay from supernatant of MPI cells pre-treated or not with acetate in the presence of absence of 2-DG 10 mM and stimulated or not with *S. pneumoniae* during 48 h. **(E)** % of intracellular viable bacteria left 6 h post infection of activated MPI cells treated or not with acetate and/or 2-DG 10 mM, normalized to vehicle group. Lines/bars showing the mean and error showing the SEM of sextuplicates **(B)** or triplicates **(C, D)** and data showing the median of triplicates **(E)**. Results are representative of three independent experiments. Statistical analysis was done using Two-way ANOVA **(B, C)** and One-Way ANOVA **(E)** corrected with Sidak’s multiple comparisons test (*p < 0.05, **p < 0.01, ***p < 0.001, ****p < 0.0001).

Glycolysis is a key metabolic pathway involved in inflammatory macrophages functions and acetate was recently described to modulate glycolysis in memory CD8^+^ T cells (38, 39). Therefore, we hypothesized that acetate could exert its effects through modulation of glycolysis in bacteria-conditioned macrophages. To test this hypothesis, we initially evaluated the effect of acetate on glycolytic activity of macrophages, by measuring the expression of key enzymes involved in glycolysis. Acetate up-regulated almost all glycolytic genes (**Figure 5A**). Glycolysis was then assessed by extracellular acidification rate (ECAR). As expected, *S. pneumoniae* promoted glycolysis in macrophages (39). Interestingly, acetate alone also boosted glycolysis and this effect was dramatically enhanced in *S. pneumoniae*-conditioned macrophages (**Figure 5B**). To assess whether glycolysis was linked to IL-1β production, macrophages were treated with acetate during glucose starvation. Glucose deprivation abrogated the capacity of acetate (and *S. pneumoniae*) to trigger IL-1β production (**Figure 5C** *left panel*). The same effect was observed when glycolysis was blocked by the glucose analogue 2-Deoxy-D-Glucose (2-DG) (**Figure 5C** *right panel*). To validate the involvement of glycolysis in NO production and bacterial killing induced by acetate, Griess and killing assays were performed in the presence of 2-DG. The inhibition of glycolysis abrogated the production of nitrite and the bacterial killing induced by acetate (**Figures 5D, E** **)**. Hence, acetate-induced glycolysis is important for IL-1β and NO production that results in an improved killing of *S. pneumoniae*.

### Acetate Increases HIF-1α Activity *Via* Glycolysis, Resulting in Higher IL-1β Production

**Figure 6:**
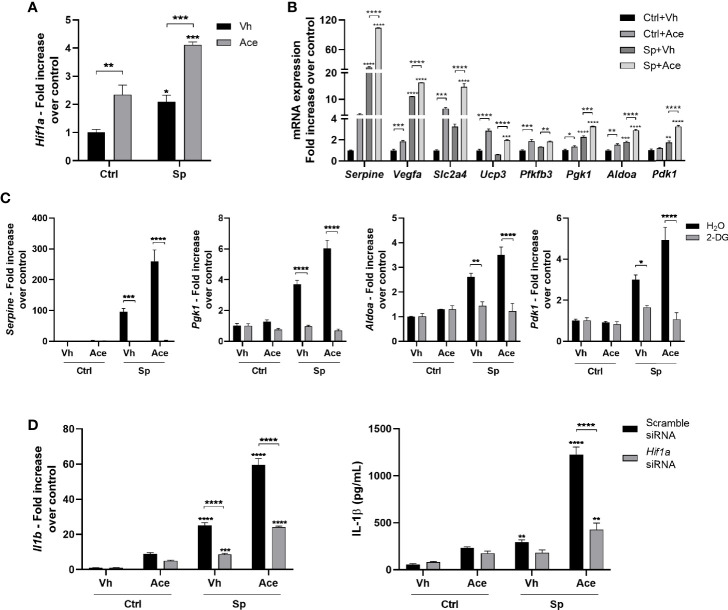
Acetate increases HIF-1α activity, *via* glycolysis, which increases IL-1β production. **(A)** Fold increase of *Hif1a* mRNA assessed by RT-PCR from MPI cells pre-treated or not with acetate and stimulated or not with *S. pneumoniae* for 18 h. Fold increase was calculated over control (Ctrl+Vh). **(B)** Fold increase from mRNA of HIF1-α target genes: plasminogen activator inhibitor 1 (*Serpine*), vascular endothelial growth factor A (*Vegfa*), Solute carrier family 2, facilitated glucose transporter member 4 (*Slc2a4*), mitochondrial uncoupling protein 3 (*Ucp3*), 6-phosphofructo-2-kinase (*Pfkfb3*), phosphoglycerate kinase 1 (*Pgk1*), fructose-bisphosphate aldolase (*Aldoa*), 3-phosphoinositide-dependent protein kinase 1 (*Pdk1*). Quantitative RT-PCR from MPI cells pre-treated or not with acetate and stimulated or not with *S. pneumoniae* for 18 h. Fold increase was calculated over each control (Ctrl+Vh). **(C)** Fold increase of *Serpine*, *Pgk1*, *Aldoa*, *Pdk1* mRNA assessed by quantitative RT-PCR from MPI cells treated or not with 2-DG and/or acetate in the presence or absence of *S. pneumoniae* for 18 h. Fold increase was calculated over control. **(D)** Fold increase of *Il1b* mRNA assessed by quantitative RT-PCR and ELISA for IL-1β from MPI cells transfected with *Hif1a* siRNA or Scramble siRNA, treated or not with acetate in the presence or absence of *S. pneumoniae* for 18 h. Fold increase was calculated over each control (Ctrl+Vh). Bars showing the mean and error showing the SEM of triplicates. Results are representative of three independent experiments. Statistical analysis was done using Two-way ANOVA corrected with Sidak’s multiple comparisons test (*p < 0.05, **p < 0.01, ***p < 0.001, ****p < 0.0001).

Gene expression profile suggested that acetate may activate the hypoxia pathway in macrophages (**Figure 1D**). Hypoxia-inducible factor-1α (HIF-1α), the main transcription factor involved in hypoxia, has been shown to be activated by glycolysis and directly induce *Il1b* transcription (40, 41). To assess the effect of acetate on HIF-1α, we evaluated HIF-1α expression and activity. Indeed, acetate, either in the presence or not of *S. pneumoniae*, promoted HIF-1α transcription in macrophages (**Figure 6A**), subsequently enhancing the expression of known HIF-1α target genes (**Figure 6B**). As glycolysis is important for HIF-1α-induced IL-1β production by macrophages (41), we hypothesized that acetate-mediated HIF-1α expression was dependent on glycolysis. After blocking glycolysis with 2-DG, acetate failed to up-regulate the expression of HIF-1α target genes, meaning that increased glycolysis mediated HIF-1α activation (**Figure 6C**). Finally, to confirm that IL-1β production was mediated through increased HIF-1α signaling in acetate-treated macrophages, we knocked-down HIF-1α (**Figure S9A**). HIF-1α silencing abrogated the capacity of acetate to modulate expression of HIF-1α target genes (**Figure S9B**). In addition, HIF-1α knock-down prevented the increase of *Il1b* gene expression and IL-1β protein production in response to acetate (**Figure 6D**). Collectively, acetate-induced glycolysis increases HIF-1α activity, and this axis is important for the production of IL-1β in *S. pneumoniae*-conditioned macrophages.

## Discussion

**Figure 7:**
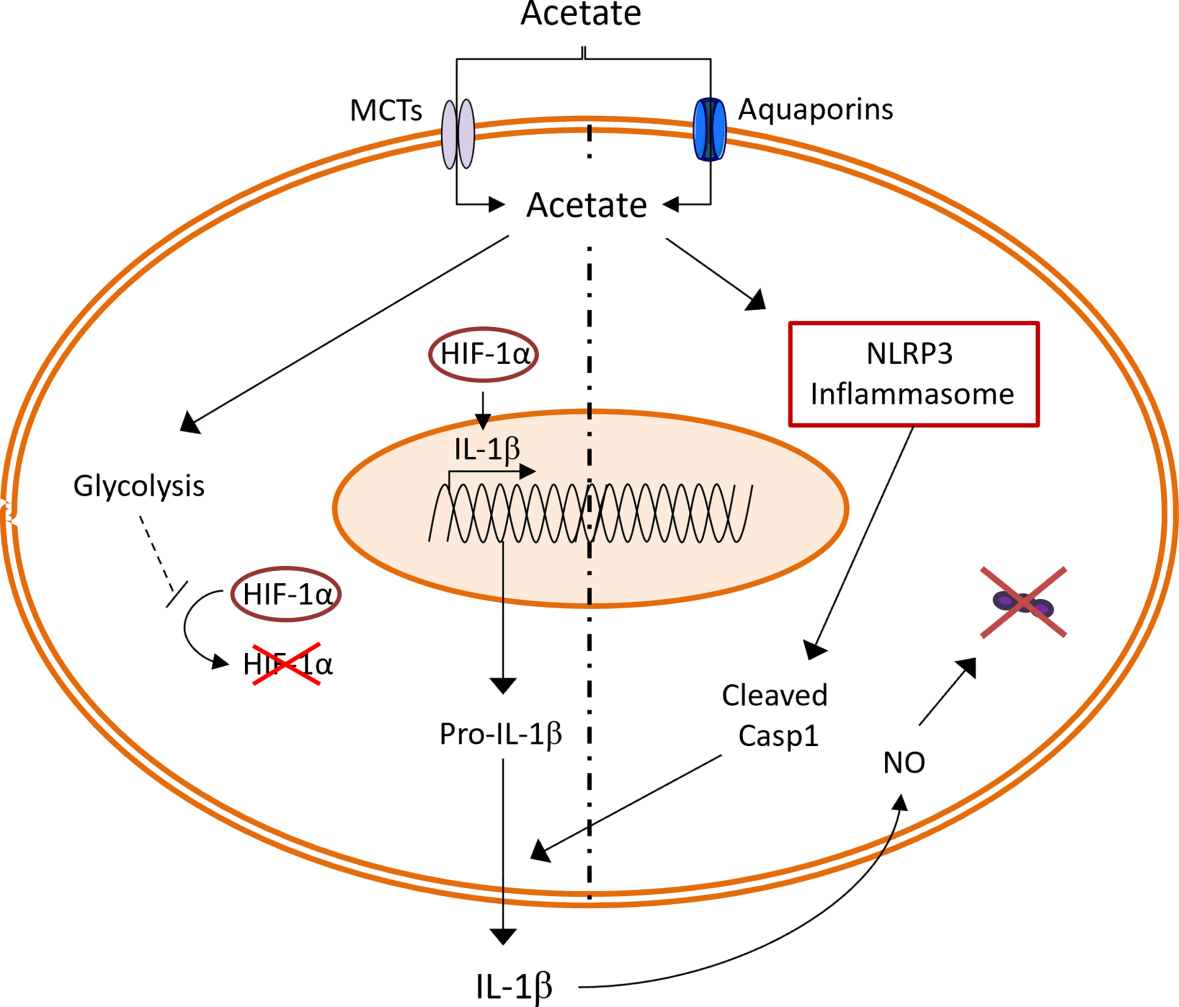
Summary of the effect of acetate on *S. pneumoniae* stimulated macrophages. Acetate increases the glycolysis, leading to higher HIF-1α activation. HIF-1α transcription factor enhance expression of *Il1b* gene. At the same time, acetate dependent on NLRP3 inflammasome increases IL-1β secretion. In an autocrine manner, IL-1β increases the production of nitric oxide (NO), which leads to *S. pneumoniae* killing.

Although SCFAs are mainly produced by the gut microbiota, acetate can also be produced by host cells during stressful conditions (3–5, 38). Emerging literature suggests that SCFAs, regardless of the source, are important in pulmonary immune defense against respiratory pathogens (9). In line with these previous findings, our group has previously shown that acetate targets alveolar macrophages to improve the clearance of *S. pneumoniae* in the context of prior influenza infection (16). In the current study, we unravel a new mechanism through which acetate boosts the bactericidal activity of alveolar macrophages. To sum up, acetate improved the glycolytic activity of macrophages leading to HIF-1α activation and increased *Il1b* transcription. In parallel, acetate relied on NLRP3 inflammasome to increase IL-1β secretion which enhanced NO production in an autocrine manner, leading to bacterial killing (**Figure 7**).

Despite the described role of SCFAs to improve the killing ability of macrophages, the mechanisms of action are only described for butyrate (16, 42–47). In line with our finding, butyrate was shown to improve the killing ability of macrophages *via* HIF-1α activation (46). However, while we propose that acetate does so by modulating glycolysis and NO production, butyrate seems to activate HIF-1α through inhibition of histone deacetylase (HDAC) leading to increased activity of lysozymes, which boost bacterial killing (46). Another study showed that butyrate inhibits HDAC3 activity, leading to reprogramming during the monocyte-to-macrophage differentiation, ultimately resulting in enhanced production of antimicrobial peptides and high bactericidal activity. (43). In this setting, butyrate increases microtubule-associated protein 1 light chain 3 alpha recruitment to the phagosome in macrophages, culminating in accelerated phagosome maturation and bacteria clearance. It is noteworthy that a mixture of SCFAs (acetate, propionate and butyrate) activates the late endosomal/lysosomal adaptor mitogen-activated protein kinase and mammalian target of rapamycin activator/regulator complex 2 (LAMTOR2) *via* FFAR2. (44). The activation of LAMTOR2 complex improves the phagosome-lysosome fusion leading to enhanced bacterial killing by alveolar macrophages. It is likely that, in this setting, acetate and/or propionate are the responsible factors as they can signal through FFAR2, unlike butyrate (16, 48). These studies highlight distinct and non-redundant mechanisms through which SCFAs (mostly butyrate) boost the bactericidal activity of macrophages. Our data showed that acetate improves the bactericidal activity of macrophages by enhancing NO production *via* glycolysis-NLRP3-IL-1β axis. Acetate-induced NO production and iNOS expression has already been demonstrated in endothelial cells and this was also linked to IL-1β production (49, 50), strongly supporting our findings. However, to the best of our knowledge, this is the first time that acetate is shown to promote NO production by bacteria-conditioned macrophages. Of note, in our condition, acetate did not induce NO production in naïve macrophages suggesting the contribution of other signaling pathways in acetate-mediated NO production by bacteria-conditioned macrophages. The effect of SCFAs on NO production by macrophages is controversial. It has been shown that acetate has no effect on NO production by macrophages upon stimulation with staphylococcal lipoprotein (51). On the other hand, another study reported that acetate decreases NO production by LPS-conditioned macrophages (52). A similar controversy exists for butyrate. In fact, butyrate decreases NO production by LPS-conditioned macrophages whilst it increases NO production by macrophages upon yeast stimulation (42, 52, 53). The reasons for these opposing findings are still unknown, but one can hypothesize that, differences in stimuli, SCFA concentrations and the cellular type (cell lines, bone marrow derived cells, *ex vivo* enriched cells) could help explain these paradoxical results.

Our study points to an important role of IL-1β in acetate-mediated NO production. Even though IFN-γ is considered the master chief cytokine to improve/promote NO production, other cytokines can play a similar role according to the context (i.e.: cellular type and stimulus) (54). Indeed, IL-17A, IL-6, TNF-α and IL-1β were shown to promote NO production in different cellular types (27, 29, 55). In line with our finding, IL-1β was also shown to increase NO production by peripheral blood mononuclear cells from healthy donors and inflammatory bowel disease patients (27). Hence, IL-1β appears to be relevant for NO production by macrophages.

The role of FFAR2 in mediating acetate-induced IL-1β production is contradictory. While FFAR2 mediates IL-1β production by neutrophils, it also attenuates IL-1β production by BMDM upon acetate treatment (31, 32). Thus, besides controversial, FFAR2 appears to be important to regulate IL-1β production. In our hands, acetate alone induced IL-1β and, in this setting, FFAR2 was critical. On the other hand, FFAR2 was dispensable for acetate-induced IL-1β production and bacterial killing by bacteria-conditioned macrophages. Similar results were observed in BMDM infected with *S. Typhimurium*, where the production of IL-1β and the bacterial clearance induced by acetate were not affected in *Ffar2* ^-/-^ BMDM (56). This suggests that, in the presence of bacteria, acetate might modulate an additional pathway that seems to be more important to IL-1β production. Besides FFAR2, acetate can enter the cells *via* MCTs and aquaporins and act as a substrate for ACSS1 or ACSS2 being converted in acetyl-CoA, which can enter the TCA cycle or be used as substrate for acetylation of histones. In the current study, we demonstrate that IL-1β production induced by acetate (in naïve and bacteria-conditioned cells) depended on MCTs. These data indicate that acetate might be acting from the intracellular compartment of the cell. However, we cannot be certain of it, because inhibition of MCTs is also described to block glycolysis and HIF-1α activation (57–59). Therefore, the decrease in IL-1β production upon addition of syrosingopine could also reflect the inhibition of glycolysis-HIF1α axis. Interestingly, the effect of acetate on IL-1β and NO production, and in the killing ability of macrophages was independent of ACSS1 and ACSS2 enzymes. To better explain how acetate can modulate IL-1β production in bacterial-conditioned macrophages, we investigated other less explored mechanisms. Acetate has been shown to activate NLRP3 inflammasome (32, 60). In line, we observed that IL-1β secretion triggered by acetate was mediated by NLRP3. Two possible hypotheses can be raised to explain how NLRP3 inflammasome might be modulating acetate-induced IL-1β secretion. First, acetate could bind directly to the NLRP3 inflammasome complex. This mechanism was recently proposed to SCFAs, where they were shown to bind to the lysine-rich region of PYRIN domain promoting the binding of ASC to NLRPs, resulting in inflammasome activation and IL-1β secretion (56). Second, acetate-induced glycolysis could be mediated by hexokinase 1, that has been shown to activate NLRP3 inflammasome and enhance IL-1β secretion by macrophages (61). Even though we could nicely demonstrate that acetate-induced glycolysis was important for HIF-1α signaling and IL-1β secretion in bacterial-conditioned macrophages, it remains elusive how NLRP3 is activated in this setting.

During metabolic stress, acetate can modulate cellular metabolism serving as an important source of acetyl-CoA. Recently, it has become clear that metabolism is important in shaping the immunophenotype of different immune cells, including macrophages (39, 62–64). Despite that, to our surprise, there are no studies describing the potential impact of acetate on macrophage’s metabolism. Here, we showed, for the first time, that acetate enhances the glycolytic activity of macrophages, and this effect is even more prominent in bacteria-conditioned cells. Such observation could be attributed to the ability of acetate to increase glyceraldehyde 3-phosphate dehydrogenase (GAPDH) activity, as shown for memory T CD8^+^ cells. Mechanistically, acetate, *via* ATP-citrate synthase activates GAPDH through acetylation (38). Consistent with the current literature, we show that acetate-induced modulation of metabolism directly impacted the immune response of macrophages (39, 41). We demonstrated that acetate-mediated glycolysis in bacterial-conditioned macrophages resulted in HIF-1α activation which promoted *Il1b* transcription. Glycolysis-driven accumulation of pyruvate has been demonstrated to inhibit prolyl hydroxylase (PHD) and, consequently, lead to HIF-1α stabilization and increased activity (40). Accordingly, LPS-induced glycolysis is important to increase succinate concentrations in inflammatory macrophages, leading to PHD inhibition and HIF-1α stabilization (41). Even though it has been suggested that butyrate, but not acetate can directly inhibit PHD activity, one can still hypothesize that acetate might indirectly modulate PHD inhibition (65). Collectively, our findings support the role of glycolysis in the acetate-mediated activation of the HIF-1α/IL-1β/NO axis and bacterial killing by macrophages.

In conclusion, our findings reinforce the concept that acetate is an important metabolite in innate immune signaling culminating in the control of bacterial intruders and identify NLRP3 and glycolysis-HIF-1α axis as critical components for bactericidal activity of macrophages.

## Data Availability Statement

The datasets presented in this study can be found in online repositories. The names of the repository/repositories and accession number(s) can be found below: https://www.ncbi.nlm.nih.gov/geo/query/acc.cgi?acc=GSE183089, GSE183089.

## Ethics Statement

All experiments complied with current national and institutional regulations and ethical guidelines (Institut Pasteur de Lille/B59-350009). The protocols were approved by the institutional ethical committee “Comité d’Ethique en Experimentation Animale” (CEEA) 75. Nord Pas-de-Calais. All experiments were approved by the “Education, Research and Innovation Ministry”, France under the registration number APAFIS22304-201910011647335v3.

## Author Contributions

MM, TP, and FT designed the experiments. MM, VS, SH, EM, and LD performed most of the experiments. MM and YR produced CRISPR Cas9 Knockout cells. BP provided antibody and inhibitors to study the inflammasome activation. MM analyzed the data. MM and FT wrote the manuscript, with input from all authors. All authors contributed to the article and approved the submitted version.

## Funding

This work was supported in part by the INSERM, CNRS, University of Lille, Pasteur Institute of Lille, and Agence Nationale de la Recherche (AAP générique 2017, ANR-17-CE15-0020-01, ACROBAT) (FT). MM and VS received salary support (PhD fellowship) by Lille University and by the Fondation pour la Recherche Médicale (VS). FT received salary support by CNRS.

## Conflict of Interest

The authors declare that the research was conducted in the absence of any commercial or financial relationships that could be construed as a potential conflict of interest.

## Publisher’s Note

All claims expressed in this article are solely those of the authors and do not necessarily represent those of their affiliated organizations, or those of the publisher, the editors and the reviewers. Any product that may be evaluated in this article, or claim that may be made by its manufacturer, is not guaranteed or endorsed by the publisher.
