# Supplementary material for: Acetate Improves the Killing of Streptococcus pneumoniae by Alveolar Macrophages via NLRP3 Inflammasome and Glycolysis-HIF-1α Axis
Source: Front Immunol. 2022 Jan 20;13:773261. doi: 10.3389/fimmu.2022.773261 (PMC8810543; doi:10.3389/fimmu.2022.773261)
Supplement: Supplementary file 1 [file DataSheet_1.pdf]

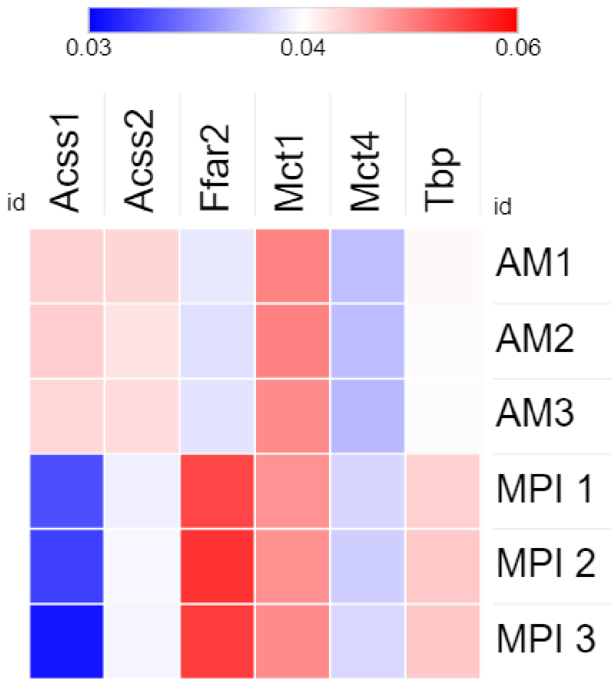

Figure S1: MPI cells and primary alveolar macrophages express all genes necessary for acetate recognition, transport, and metabolism.

mRNA was extracted from MPI cells and primary alveolar macrophages to quantify gene expression of Acetyl-coenzyme A synthetase (*Acss*), Free fatty acid receptor 2 (*Ffar2*), Monocarboxylate transporters (*Mct*) and the house keeping TATA-box binding protein (*Tbp*) by RT-PCR. The presented heatmap is a result of the calculation: 1/ct. Ct of 17 was considered as the maximal expression, being 0.058 and Ct of 40 was considered as the minimal expression, being 0.025.

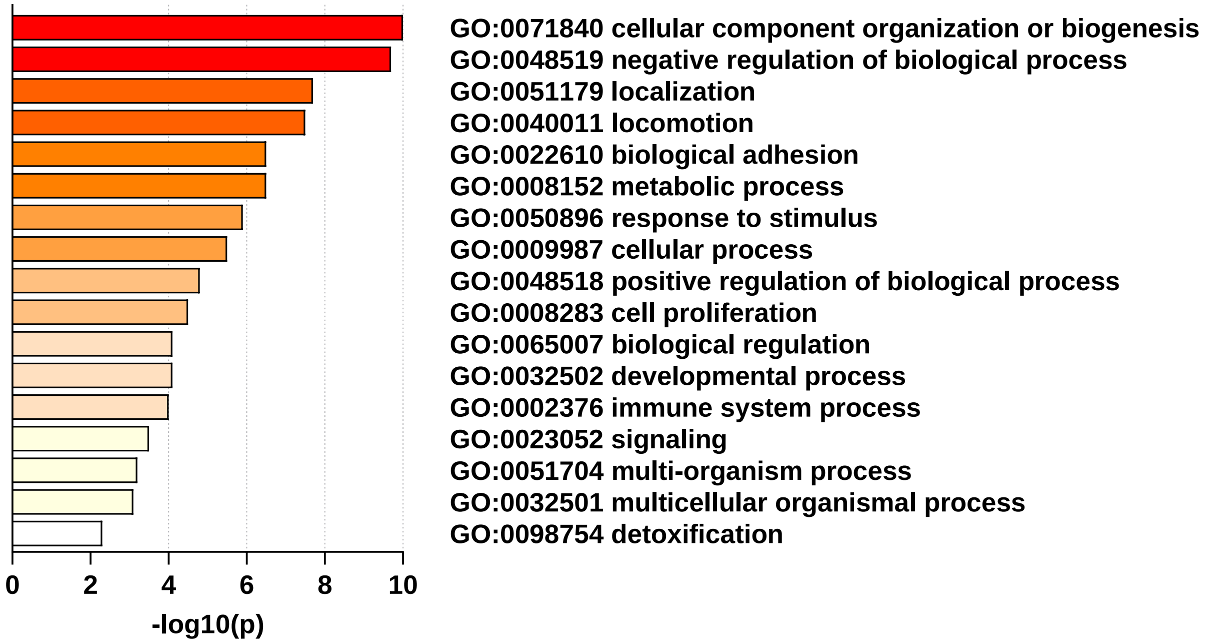

Figure S2: Acetate modulates macrophage's profile.

Overview of all pathways modulates by acetate. Enrichment analysis from MPI cells pre-treated or not with acetate, stimulated with *S. pneumoniae* and after 18h lysed for RNA extraction and sequencing. Enrichment analysis was done over untreated stimulated cells in the Metascape platform. Genes with log2 fold change > 0.6 and p-adjunctive < 0.05 were used for the enrichment analysis.

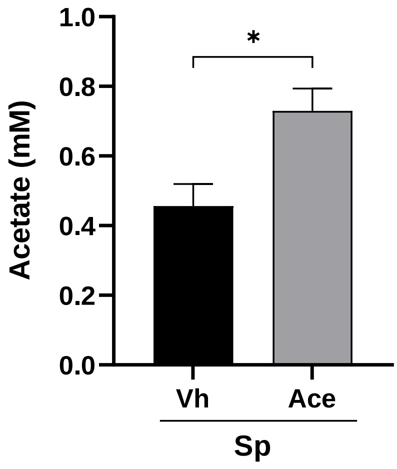

Figure S3: Acetate quantification on conditioned medium.

Acetate quantification from supernatant of MPI cells pre-treated or not with acetate and stimulated or not with *S. pneumoniae* for 24 h. Bars showing the mean and error showing the SEM of triplicates. Statistical analysis was done using Unpaired Student’s t-test (*$p < 0.05$).

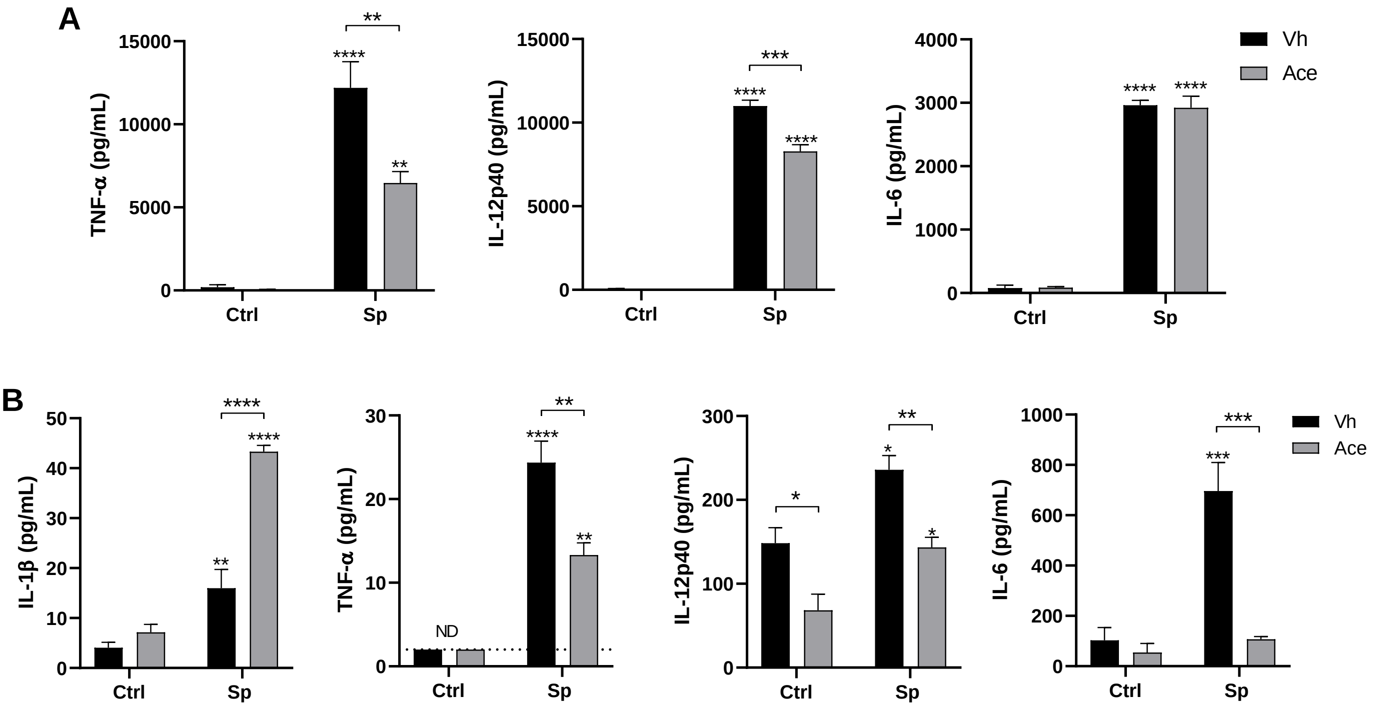

Figure S4: MPI cells resemble alveolar macrophages and have similar secretion of cytokines in response to acetate.

(A) ELISA for TNF-α, IL-12p40 and IL-6 from supernatant of MPI cells pre-treated or not with acetate and stimulated or not with *S. pneumoniae* for 24 h.

(B) ELISA for IL-1β, TNF-α, IL-12p40 and IL-6 from supernatant of alveolar macrophages pre-treated or not with acetate and stimulated or not with *S. pneumoniae* for 24 h.

Bars showing the mean and error showing the SEM of triplicates. Results are representative of three independent experiments. Statistical analysis was done using Two-way ANOVA corrected with Sidak's multiple comparisons test (*$p < 0.05$, **$p < 0.01$, ***$p < 0.001$, ****$p < 0.0001$).

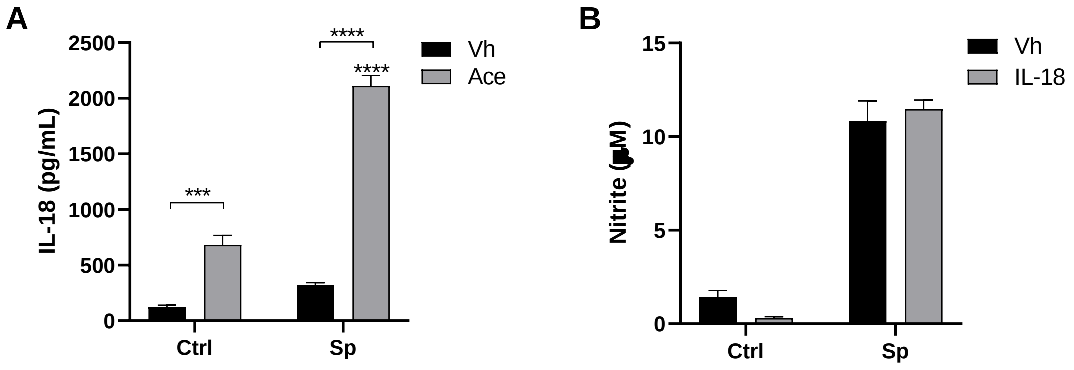

Figure S5: Acetate enhances IL-18 production, but IL-18 does not impact on NO production.

(A) ELISA of IL-18 from supernatant of MPI cells pre-treated or not with acetate and stimulated or not with *S. pneumoniae* during 24 h.

(B) Nitrite concentration assessed by Griess assay from supernatant of MPI cells pre-treated or not with recombinant IL-18 during 48 h.

Bars showing the mean and error showing the SEM of triplicates. Results are representative of three independent experiments. Statistical analysis was done using Two-way ANOVA corrected with Sidak's multiple comparisons test (***$p < 0.001$, ****$p < 0.0001$).

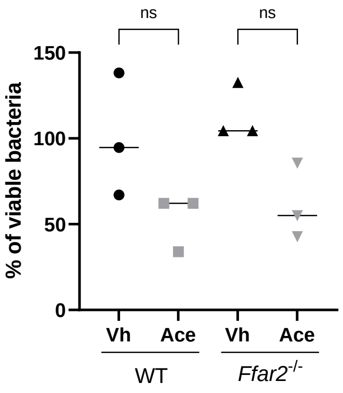

Figure S6: Acetate seems to be independent of FFAR2 to induce bacterial killing.

% of intracellular viable bacteria left 6 h post infection of activated WT and *Ffar2*$^{-/-}$ primary alveolar macrophages treated or not with acetate, normalized to vehicle group.

Data showing the median of triplicates. Statistical analysis was done using One-Way ANOVA corrected with Sidak's multiple comparisons test.

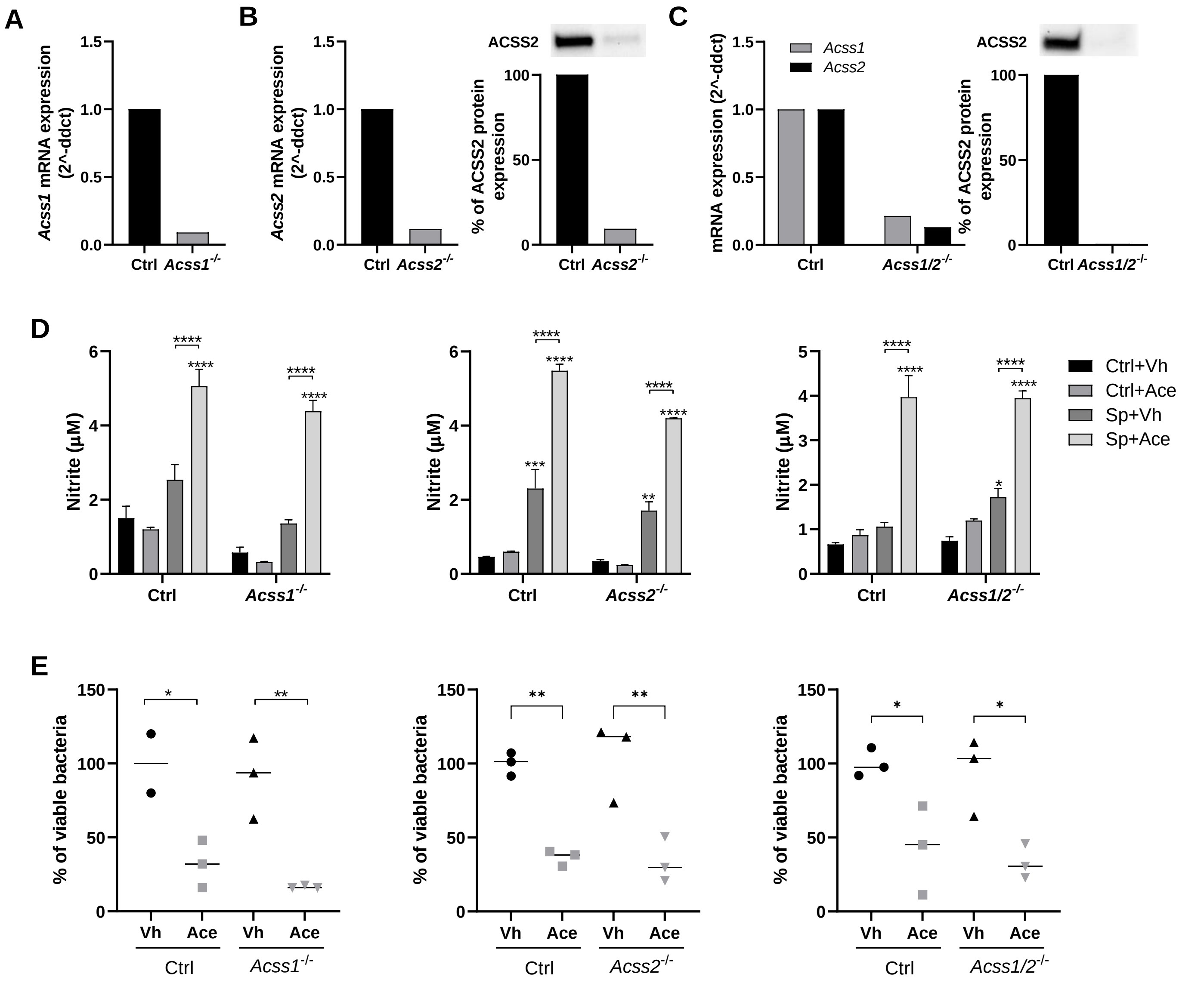

Figure S7: Acetate does not depend on ACSSs to increase NO production and boost macrophage's killing ability.

(**A-C**) ACSS1 and ACSS2 genes were knockout from MPI cells with CRISPR Cas9 (lentivirus). Cells were transduced with lentiviruses and submitted to antibiotic selection. Later, the best KOs were selected to be used further. **(A** and **C)** Fold change of *Acss1* mRNA expression assessed by RT-PCR from MPI control, *Acss1*$^{-/-}$ and *Acss1/2*$^{-/-}$ cells. ACSS1 protein was undetectable by western blot. **(B** and **C)** Fold change of *Acss2* mRNA expression assessed by RT-PCR and ACSS2 protein assessed by western blot from MPI control, *Acss2*$^{-/-}$ and *Acss1/2*$^{-/-}$ cells.

(D) Nitrite concentration assessed by Griess assay from supernatant of MPI control, *Acss1*$^{-/-}$, *Acss2*$^{-/-}$ and *Acss1/2*$^{-/-}$ cells pre-treated or not with acetate and stimulated or not with *S. pneumoniae* during 48 h.

(E) % of intracellular viable bacteria left 6 h post infection of activated MPI control, *Acss1*$^{-/-}$, *Acss2*$^{-/-}$ and *Acss1/2*$^{-/-}$ cells treated or not with acetate, normalized to vehicle group.

Bars showing the mean and error showing the SEM **(A-D)** or data showing the median **(E)** of triplicates. Results are representative of three independent experiments. Statistical analysis was done using Two-way ANOVA **(D)** and One-Way ANOVA **(E)** corrected with Sidak's multiple comparisons test (*p < 0.05, **p < 0.01, ***p < 0.001, ****p < 0.0001).

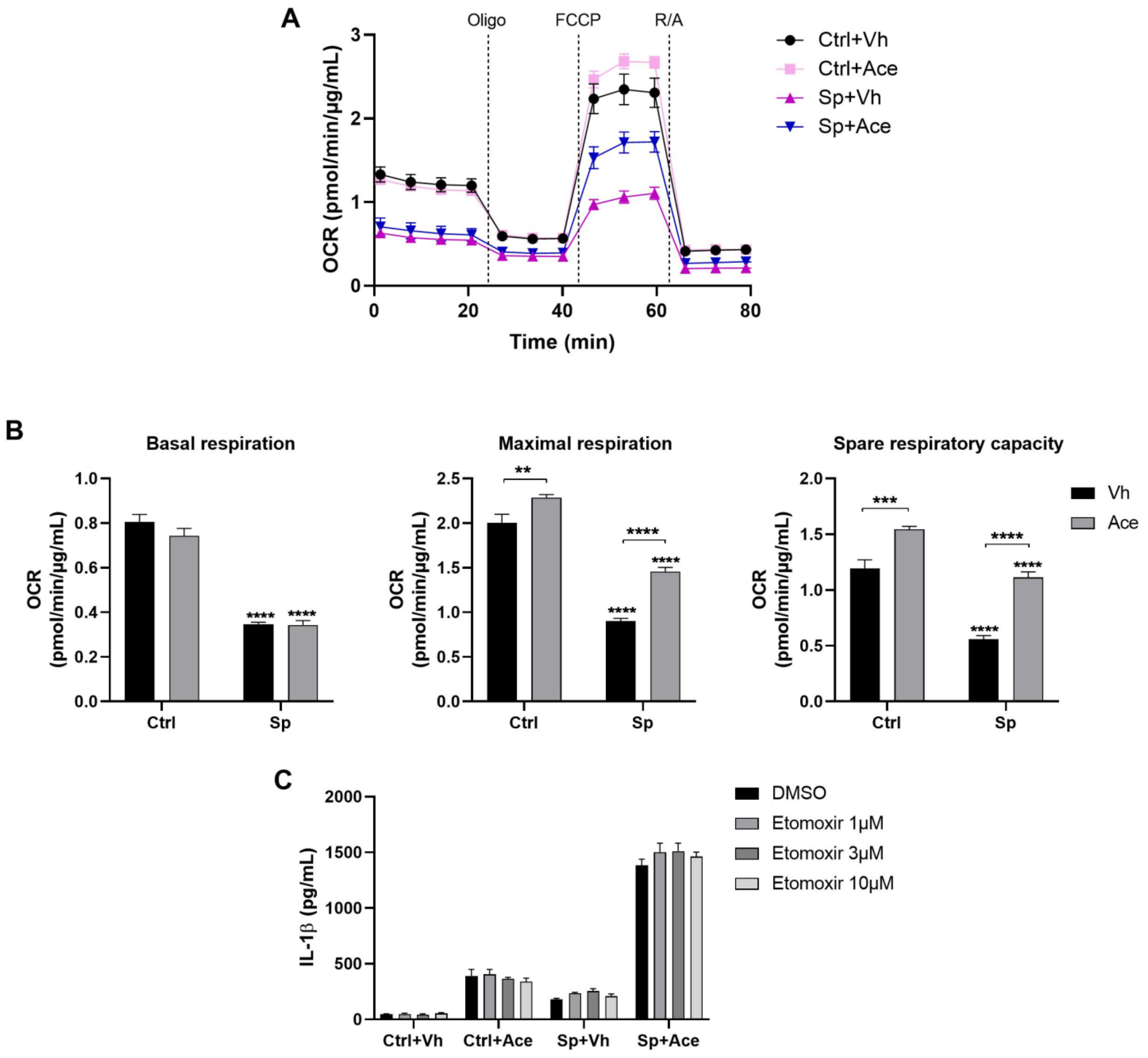

Figure S8: Acetate modulates oxygen consumption of macrophages

(A and **B)** Mitochondrial stress assay of MPI cells pre-treated or not with acetate, and then stimulated or not with *S. pneumoniae* for 24 h. OCR was measured in Seahorse after injection of oligomycin (Oligo), FCCP and Rotenone and Antimycin (R/A). Basal respiration **(left *panel*)** was calculated by the subtraction of the lower measurement after R/A injection from the last measurement of basal respiration. Maximal respiration **(*middle panel*)** by the subtraction of the lower measurement after R/A injection from the maximal measurement after FCCP injection. Spare respiratory capacity **(*right panel*)** by the subtraction of the lower measurement after R/A injection, and the last measurement of basal respiration from the maximal measurement after FCCP injection.

(C) ELISA for IL-1β from supernatant of MPI cells pre-treated or not with acetate in the presence or absence of etomoxir and then stimulated or not with *S. pneumoniae* for 24 h.

Lines/bars showing the mean and error showing the SEM of sextuplicates **(A** and **B)** or triplicates **(C)**. Results are representative of three independent experiments. Statistical analysis was done using Two-way ANOVA corrected with Sidak's multiple comparisons test (**$p < 0.01$, ****$p < 0.0001$).

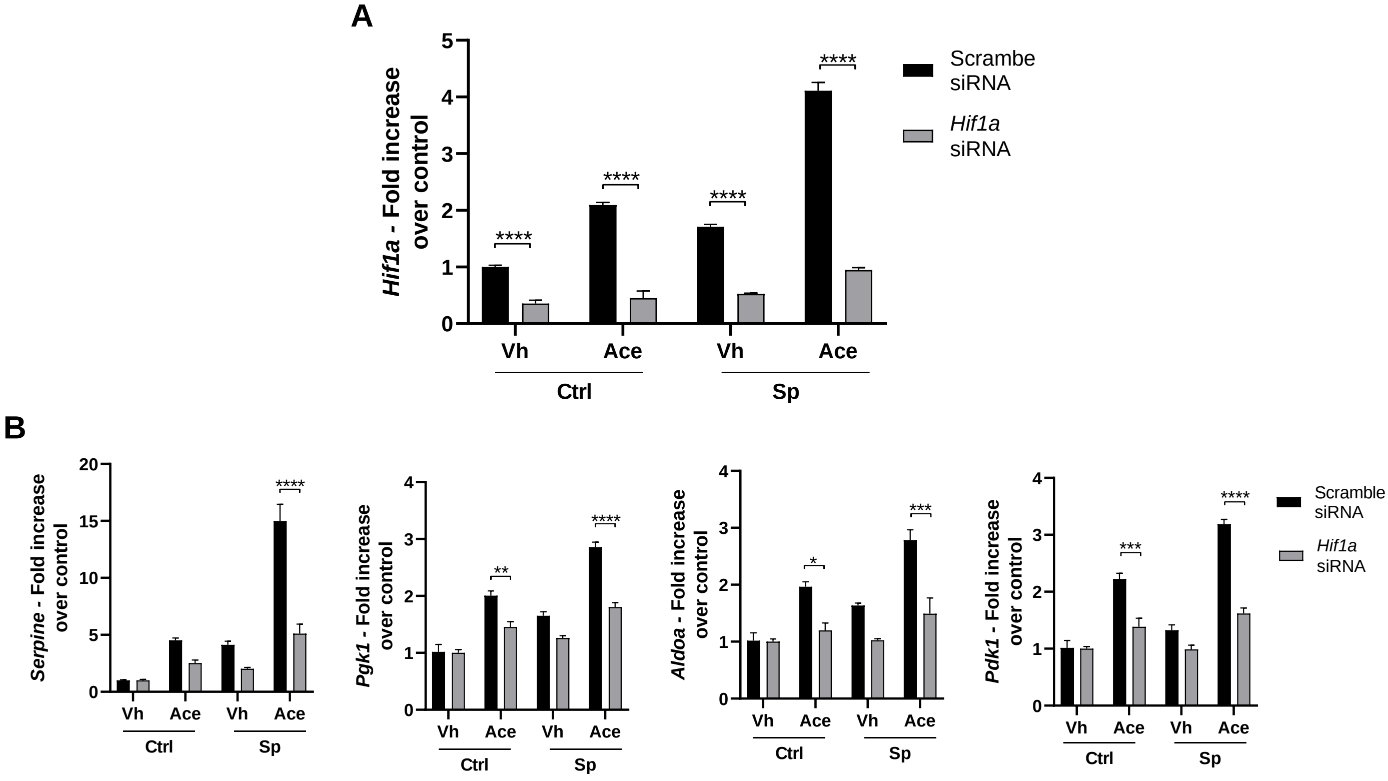

Figure S9: HIF-1α knockdown cells.

(A) Fold increase of *Hif1a* mRNA assessed by RT-PCR from scramble siRNA or *Hif1a* siRNA transfected cells treated or not with acetate in the presence or absence of *S. pneumoniae* for 18 h. Fold increase was calculated over scramble siRNA control (Ctrl+Vh).

(B) Fold increase of *Serpine*, *Pgk1*, *Aldoa*, *Pdk1* mRNA assessed by RT-PCR from scramble siRNA or *Hif1a* siRNA transfected cells treated or not with acetate in the presence or absence of *S. pneumoniae* for 18 h. Fold increase was calculated over each control (Ctrl+Vh).

Bars showing the mean and error showing the SEM of triplicates. Results are representative of three independent experiments. Statistical analysis was done using Two-way ANOVA corrected with Sidak's multiple comparisons test (**p < 0.01, ****p < 0.0001).
